# Computer self-efficacy and perceived knowledge application in generative AI-supported learning: a cross-sectional parallel mediation analysis

**DOI:** 10.3389/fpsyg.2026.1927402

**Published:** 2026-08-26

**Authors:** Yan Cheng, Haibo Liu

**Affiliations:** 1 School of General Education, Shandong Huayu University of Technology, Dezhou, China; 2 Dezhou Information Engineering Secondary Vocational School, Dezhou, China

**Keywords:** autonomous learning technology use, computer self-efficacy, generative artificial intelligence, higher education, human–AI synergy, parallel mediation, perceived knowledge application

## Abstract

**Background:**

This study examined whether human–AI synergy (HAS) and autonomous learning technology use (ALT) mediated the association between computer self-efficacy (CSE) and perceived knowledge application ability (KAA).

**Methods:**

A cross-sectional survey of 801 undergraduates from five higher education institutions in Shandong Province, China, was analyzed using confirmatory factor analysis, structural equation modeling, and bootstrapping.

**Results:**

CSE was positively associated with HAS and ALT. ALT showed a positive indirect association with perceived KAA, whereas HAS showed a negative conditional indirect association. The direct CSE–KAA association was not significant after including the mediators.

**Conclusion:**

CSE was associated with perceived knowledge application through two opposite indirect pathways. The negative HAS coefficient should be interpreted cautiously because HAS measured collaboration rather than cognitive offloading, and KAA was self-reported.

## 1 Introduction

The rapid diffusion of generative artificial intelligence (GAI) is reshaping the learning ecology of higher education. Unlike earlier educational technologies that primarily supported information retrieval, content presentation, or learning management, contemporary GAI systems can generate explanations, summarize texts, provide real-time feedback, assist with problem solving, and sustain dialogic interaction with learners (Bearman et al., 2023; Crompton and Burke, 2023). These affordances have expanded the role of digital technology from a supplementary resource to a potential partner in academic writing, programming, language learning, disciplinary inquiry, and project-based learning (Cena et al., 2026; Wang et al., 2026).

The educational value of GAI, however, cannot be understood solely in terms of access or frequency of use. AI-supported learning can facilitate resource searching, explanation, feedback, idea generation, and task planning, but the same tools can also be used in ways that reduce learners’ monitoring, verification, or independent cognitive engagement. Research has therefore increasingly emphasized the conditions under which learners regulate AI assistance, evaluate generated content, and retain responsibility for decisions and final outputs (Fan et al., 2025; Hou et al., 2025; Wong and Qiu, 2026). Recent work on AI-supported self-regulated learning, learner autonomy, and AI metacognition further suggests that students’ strategic regulation of AI use may be as important as the availability of the technology itself (He, 2025; El Hosayny et al., 2026; Wu et al., 2026).

This issue is particularly relevant to knowledge application. Higher education aims not only to support the acquisition of information but also to help students transfer concepts, principles, and methods to unfamiliar situations and practical problems. Knowledge application corresponds to a higher-order level of learning that requires retrieval, reorganization, evaluation, and contextual adaptation (Krathwohl, 2002). In AI-rich environments, successful completion of a task with technological support does not necessarily demonstrate what a student can independently apply. The present study therefore focuses specifically on perceived knowledge application ability: students’ self-reported judgments about their ability to use newly acquired knowledge in academic and practical contexts. This construct provides insight into learners’ perceived competence, but it is not equivalent to an objective performance assessment.

Computer self-efficacy is a relevant psychological antecedent of technology-supported learning. It refers to individuals’ beliefs about their capability to use computer-based or digital technologies to accomplish tasks (Compeau and Higgins, 1995). Students with stronger computer self-efficacy are generally more willing to explore unfamiliar tools, persist through technical difficulty, and integrate digital resources into their learning activities. In GAI-supported contexts, such confidence may influence whether students experiment with prompts, compare generated responses, request feedback, and incorporate AI tools into learning routines (Alghamdi, 2025; Lin and Wang, 2025). Nevertheless, efficacy beliefs do not guarantee particular learning outcomes; their educational relevance depends on the behaviors through which they are enacted.

Existing research often treats AI use as a broad or homogeneous construct. This approach can obscure meaningful differences between forms of engagement. Human-AI synergy emphasizes interactive and collaborative activity, such as iterative prompting, feedback exchange, explanation generation, and joint problem solving (Nguyen et al., 2024; Hwang and Lee, 2025). Autonomous learning technology use emphasizes learners’ self-directed use of technology to plan, monitor, organize, and support their learning while retaining control over goals and decisions (Teo et al., 2010; Lin et al., 2025). These behaviors may coexist, but they are conceptually distinct. Interactive collaboration is not inherently equivalent to dependence, and autonomous use does not mean learning without assistance.

The present study examines whether HAS and ALT operate as parallel mediators in the association between CSE and perceived KAA. This framing differs from the stronger claim that HAS directly measures cognitive offloading. Cognitive offloading concerns the use of external resources to reduce internal cognitive effort, whereas the HAS items in this study assess perceived collaboration and task coordination. A negative conditional coefficient for HAS may be compatible with several explanations, including suppression among correlated predictors, reverse selection into AI support, unmeasured metacognitive differences, or forms of dependence not captured by the present measure. Accordingly, cognitive offloading is treated only as a possible topic for future direct investigation, not as a construct demonstrated by the current data (Fan et al., 2025; Zahid et al., 2026).

This study makes three contributions. First, it extends research on CSE beyond technology acceptance by examining its association with perceived knowledge application (Venkatesh et al., 2012). Second, it distinguishes HAS from ALT as two forms of AI-related learning behavior within a parallel mediation framework. Third, it provides a cautious account of opposite-direction indirect associations while explicitly recognizing the limits of cross-sectional self-report data. This approach supports a more differentiated understanding of GAI-supported learning without treating collaboration, autonomy, dependence, and cognitive offloading as interchangeable concepts.

## 2 Theoretical foundations and literature review

### 2.1 Social cognitive theory and technology-mediated learning

Social Cognitive Theory explains human functioning through reciprocal relations among personal factors, behavior, and environmental conditions (Bandura, 1986). Learners do not simply respond to technological resources; they interpret and select them according to perceived capabilities, goals, expectations, and self-regulatory processes. In GAI-supported learning, the same technological environment may therefore produce different patterns of engagement because students differ in their confidence, strategies, monitoring, and willingness to assume responsibility for learning decisions.

Self-efficacy is central to this framework because it influences task initiation, persistence, strategy selection, and responses to difficulty. GAI-supported tasks often require learners to formulate prompts, evaluate generated information, manage uncertainty, identify errors, and integrate external suggestions with prior knowledge. Students who feel capable of using digital systems may engage more actively with these functions. At the same time, Social Cognitive Theory does not imply that confidence alone produces learning outcomes. Efficacy beliefs affect outcomes through behaviors and regulatory processes, making AI-related learning behaviors plausible mediators between CSE and perceived KAA.

Self-regulated learning and AI metacognition provide complementary perspectives. Strategic AI use involves setting goals, deciding when assistance is needed, monitoring the reliability of outputs, and revising strategies in response to feedback. Research on learner autonomy and AI-supported regulation suggests that metacognitive engagement can shape how technological confidence is translated into learning activity and perceived competence (He, 2025; El Hosayny et al., 2026; Wu et al., 2026). These perspectives support examining more than one behavioral pathway rather than assuming a uniform effect of AI use.

### 2.2 Computer self-efficacy and perceived knowledge application ability

CSE refers to individuals’ beliefs that they can successfully use computer-based or digital technologies to complete relevant tasks (Compeau and Higgins, 1995). In educational settings, students with higher CSE are more likely to explore digital tools, solve technical problems, and use technological resources in academic work. In GAI-supported learning, CSE may also increase students’ confidence in interacting with intelligent systems, evaluating outputs, and adapting technological functions to learning goals (Alghamdi, 2025; Lin and Wang, 2025).

Perceived KAA refers in this study to students’ self-reported ability to transfer and use newly acquired knowledge in academic tasks or practical problem solving. Students with stronger CSE may perceive greater access to resources, more control over technology-supported inquiry, and greater capability to test alternative solutions. These experiences may be positively associated with perceived KAA. However, because the outcome is self-reported and the design is cross-sectional, the hypothesis concerns an association rather than a demonstrated causal effect.

*H1*: Computer self-efficacy has a positive direct association with perceived knowledge application ability after accounting for HAS and ALT.

### 2.3 Computer self-efficacy and human-AI synergy

HAS refers to learners’ perceived engagement in interactive, complementary, and collaborative processes with AI systems. It includes activities such as multi-turn dialogue, iterative prompting, feedback seeking, explanation generation, revision, and problem-solving support (Nguyen et al., 2024; Hwang and Lee, 2025). The construct describes the extent of collaboration, not whether that collaboration is productive, dependent, or cognitively substitutive.

Students with stronger CSE may be more willing to initiate and sustain complex AI interaction. They may feel more capable of reformulating prompts, comparing outputs, identifying limitations, and coordinating task contributions between themselves and an AI system. Lower CSE may instead be accompanied by avoidance, anxiety, or limited use of advanced functions. Therefore, CSE is expected to be positively associated with HAS.

*H2*: Computer self-efficacy is positively associated with human-AI synergy.

### 2.4 Computer self-efficacy and autonomous learning technology use

ALT refers to students’ self-directed use of digital or AI-based tools to plan, monitor, regulate, and support learning. It emphasizes learners’ active control over goals, schedules, resource selection, progress monitoring, and decisions about when and how to use technological support (Teo et al., 2010; Li and Fu, 2024). AI functions as an external resource, while the learner remains responsible for the organization and regulation of the learning process.

CSE may support ALT by increasing perceived control over technology-mediated learning. Students who believe they can use digital tools effectively may be more likely to integrate them into learning routines, persist when technical problems arise, and select functions that match specific goals. In GAI-supported settings, this may include using AI to locate resources, obtain supplementary explanations, check understanding, or monitor progress while retaining responsibility for final judgments.

*H3*: Computer self-efficacy is positively associated with autonomous learning technology use.

### 2.5 Human-AI synergy and perceived knowledge application

Interactive collaboration with AI can potentially support learning by providing immediate explanations, alternative perspectives, feedback, and opportunities for iterative refinement. When learners critically evaluate and transform AI-generated information, HAS may stimulate reflection and support knowledge construction (Hou et al., 2025; Wong and Qiu, 2026). Human-AI collaboration should therefore not be assumed to be inherently harmful.

At the same time, the association between HAS and perceived KAA may depend on how collaboration is regulated. Students may differ in whether they challenge AI outputs, verify information, articulate their own reasoning, or delegate core task processes. Research on metacognitive laziness and AI reliance suggests that intensive interaction can coexist with reduced cognitive monitoring under some conditions (Fan et al., 2025; Zahid et al., 2026). However, these conditions were not directly measured by the HAS scale used here. The scale captures perceived task division and collaboration, not excessive reliance or cognitive offloading.

Because the theoretical literature permits both supportive and adverse interpretations, and because the present measure cannot distinguish productive from dependent synergy, a non-directional mediation hypothesis is more appropriate than defining HAS in advance as a cognitive-offloading pathway. The empirical direction of the indirect association is therefore examined without equating a negative coefficient with a directly measured mechanism.

*H4*: Human-AI synergy mediates the association between computer self-efficacy and perceived knowledge application ability.

### 2.6 Autonomous learning technology use and perceived knowledge application

ALT may support perceived KAA by helping students obtain and organize learning resources, identify knowledge gaps, monitor understanding, and connect abstract concepts with practical examples. These activities involve active selection, evaluation, and integration rather than passive receipt of information. Self-directed technology use may therefore provide more opportunities to rehearse and apply knowledge in ways that strengthen students’ perceptions of their own capability (Lin et al., 2025; Ge and Li, 2026).

Within Social Cognitive Theory, ALT represents a behavioral pathway through which efficacy beliefs can be enacted. Students with stronger CSE may be more likely to use AI strategically as part of a self-regulated learning process, and this pattern of use may be positively associated with perceived KAA.

*H5*: Autonomous learning technology use positively mediates the association between computer self-efficacy and perceived knowledge application ability.

### 2.7 Parallel mediation framework

CSE may be associated simultaneously with HAS and ALT. Both behaviors involve AI-supported learning, but they emphasize different aspects of engagement: HAS concerns interaction and coordination with AI, whereas ALT concerns self-directed regulation of technology use. A parallel mediation model allows the two indirect associations to be estimated while each mediator is adjusted for the other.

The model does not assume that the mediators represent mutually exclusive categories or that their coefficients identify causal mechanisms. Instead, it tests whether CSE is associated with perceived KAA through statistically distinct pathways. Opposite signs, if observed, indicate inconsistent or competitive indirect associations in the statistical sense described in mediation research, but they do not by themselves establish cognitive offloading, dependence, or temporal precedence (Zhao et al., 2010).

*H6*: Human-AI synergy and autonomous learning technology use jointly operate as parallel mediators in the association between computer self-efficacy and perceived knowledge application ability.

**Figure 1:**
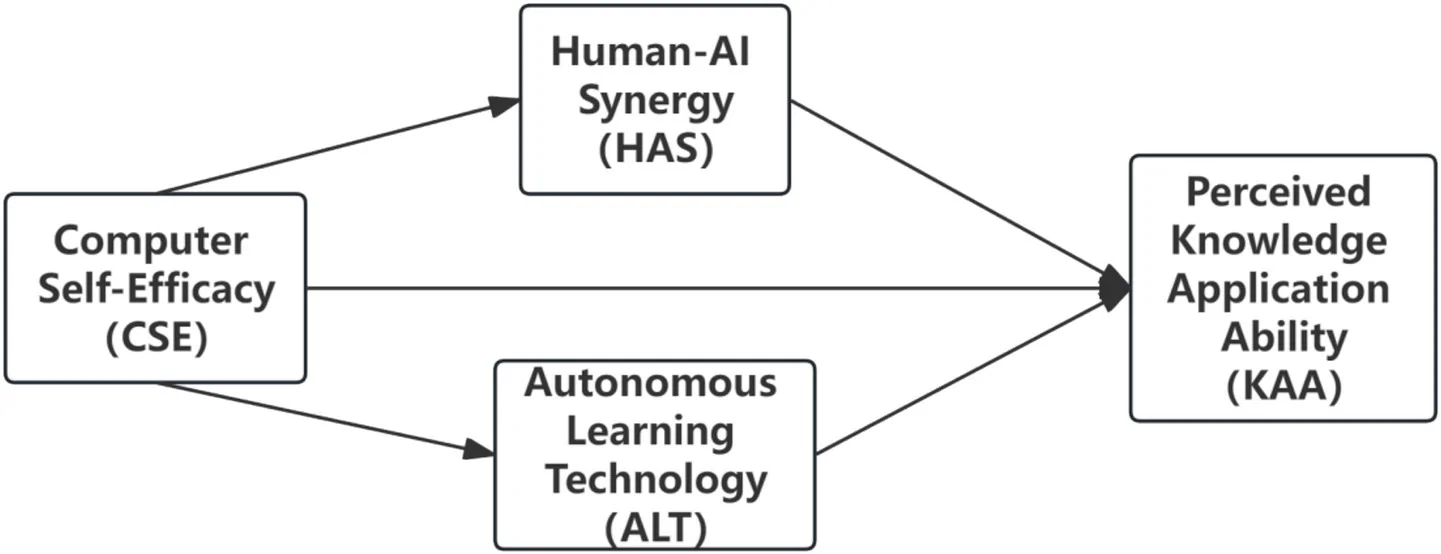
Conceptual model and hypothesized paths of the current study.

The conceptual model is presented in Figure 1.

## 3 Materials and methods

### 3.1 Participants and data collection

This study employed a cross-sectional online survey design. Data were collected between February and April 2026 from undergraduate students at five higher education institutions in Shandong Province, China. Convenience and snowball sampling were used to recruit participants. The electronic questionnaire was distributed through university student communities, WeChat class groups, and academic forums. Participation was voluntary and anonymous. Before completing the questionnaire, all participants were informed of the purpose of the study, the voluntary nature of participation, the confidentiality and anonymity of their responses, and their right to withdraw at any time. Electronic written informed consent was obtained from all participants before questionnaire completion.

The questionnaire was administered in a fixed sequence. Participants first completed questions concerning demographic characteristics and generative AI use, followed by the perceived knowledge application ability (KAA), human–AI synergy (HAS), computer self-efficacy (CSE), and autonomous learning technology use (ALT) scales. Neither the scale blocks nor the individual items were randomized or counterbalanced.

The online questionnaire required participants to answer all mandatory items before submission. Consequently, the submitted questionnaires contained no item-level missing values. A total of 900 questionnaires were submitted. Response-quality screening was conducted sequentially using three prespecified criteria. First, 52 questionnaires were excluded because the completion time was less than 90 s. Second, 31 questionnaires were excluded because they showed obvious straight-lining or patterned responding. Third, 16 questionnaires were excluded because the respondents failed the embedded attention-check items. Each questionnaire was counted only once according to the first exclusion criterion it met. In total, 99 questionnaires were excluded, leaving 801 valid questionnaires for analysis. The resulting valid-questionnaire retention rate was 89.0%. Because the number of students who received or viewed the survey invitation was unknown, this percentage represents a questionnaire-retention rate rather than a response rate.

The final sample included 371 male students (46.3%) and 430 female students (53.7%). Participants represented different age groups, academic years, and academic disciplines and reported using different generative AI tools with varying frequencies and lengths of experience. Detailed participant characteristics are presented in Table 1.

**Table 1:** Demographic characteristics and generative AI use of participants.

Variable	Category	*n*	%
Gender	Male	371	46.3
	Female	430	53.7
Age	18 years or younger	92	11.5
	19 years	205	25.6
	20 years	221	27.6
	21 years	176	22.0
	22 years or older	107	13.4
Academic year	First year	213	26.6
	Second year	231	28.8
	Third year	206	25.7
	Fourth year	151	18.9
Academic discipline	Humanities and social sciences	186	23.2
	Education	132	16.5
	Business and economics	149	18.6
	Science, technology, engineering, and mathematics	214	26.7
	Arts and other disciplines	120	15.0
Primary generative AI tool	DeepSeek	224	28.0
	ChatGPT	173	21.6
	Doubao	126	15.7
	Kimi	114	14.2
	Qwen	91	11.4
	ERNIE Bot	73	9.1
Frequency of generative AI use	Daily	179	22.3
	Three to five times per week	248	31.0
	One to two times per week	236	29.5
	Less than once per week	138	17.2
Experience using generative AI	Less than 6 months	177	22.1
	6–12 months	245	30.6
	1–2 years	265	33.1
	More than 2 years	114	14.2

### 3.2 Measurement instruments

The four constructs were measured using scales adapted from previous research and contextualized for generative AI-supported learning. For instruments originally developed in English, translation and back-translation procedures were used to ensure semantic equivalence (Brislin, 1970). Two experts in educational technology reviewed the adapted items for face and content validity. Minor wording revisions were subsequently made based on expert feedback and pilot testing. All items were rated on a five-point Likert scale ranging from 1 (strongly disagree) to 5 (strongly agree), with higher scores indicating higher levels of the corresponding construct.

Computer self-efficacy. Computer self-efficacy (CSE) was measured using items adapted from research on technology-related self-efficacy and AI tool use (Fang et al., 2024). The initial scale contained three items. During measurement evaluation, one item was removed because it did not demonstrate adequate psychometric performance in the measurement model. The final CSE scale therefore contained two items assessing students’ perceived competence in using digital or AI tools for learning. A representative item was: “I am confident in independently solving technical problems encountered when using AI tools for learning.”

Human–AI synergy. Human–AI synergy (HAS) was measured using three items adapted from research on human–machine collaboration in AI application scenarios (Li et al., 2023). The items assessed students’ perceived engagement in interactive, complementary, and collaborative processes with AI during learning. A representative item was: “When completing complex learning tasks, I can form effective task division and collaboration with AI tools.” The scale assessed perceived collaboration and task coordination rather than cognitive offloading, excessive dependence, or uncritical acceptance of AI-generated information.

Autonomous learning technology use. Autonomous learning technology use (ALT) was measured using three items adapted from the Self-Directed Learning with Technology Scale (SDLTS; Teo et al., 2010). The items assessed students’ tendency to use digital or AI-based tools to plan, monitor, organize, and manage their learning. A representative item was: “I actively use AI technology to plan and manage my extracurricular learning progress.”

Perceived knowledge application ability. Perceived knowledge application ability (KAA) was measured using four items adapted from student learning outcome assessments emphasizing knowledge transfer and practical application (Lo et al., 2022). The items assessed students’ self-reported ability to apply newly acquired knowledge to academic tasks and practical problem-solving situations. A representative item was: “I can flexibly apply new knowledge acquired through AI assistance to actual course projects or problem solving.” The measure reflected students’ perceived knowledge application ability rather than objectively assessed knowledge-transfer performance.

The final measurement model therefore consisted of 12 observed items: two CSE items, three HAS items, three ALT items, and four perceived KAA items. The complete measurement items, their sources, and item-retention status are presented in Supplementary Table S1.

### 3.3 Common method Bias control

Because all variables were measured using a self-reported questionnaire administered at a single time point, common method bias was a potential concern. Procedural measures included anonymous participation, assurances of response confidentiality, and an introductory statement emphasizing that there were no right or wrong answers. The questionnaire nevertheless followed a fixed sequence consisting of demographic and generative AI-use questions, followed by the KAA, HAS, CSE, and ALT scales; neither the scale blocks nor the individual items were randomized or counterbalanced (Podsakoff et al., 2003).

Common method bias was further assessed statistically. First, Harman’s single-factor test was conducted to determine whether one factor accounted for most of the total variance. Second, a single-factor confirmatory factor analysis model was compared with the hypothesized four-factor measurement model. Third, an unmeasured latent method construct was added to the measurement model to examine whether the inclusion of a common method factor substantially improved model fit or changed the interpretation of the substantive relationships (Liang et al., 2007). The results of these analyses are reported in Section 4.1.

### 3.4 Data analysis strategy

Data analysis was conducted using SPSS 26.0 and AMOS 24.0. The analysis proceeded in several stages. First, the submitted questionnaires were screened for invalid responses, missing values, and potential outliers. The response-quality criteria and exclusion process are described in Section 3.1. Because the online questionnaire required responses to all mandatory items before submission, no item-level missing values were present in the 801 retained questionnaires. Therefore, no missing-data imputation was required.

Potential univariate outliers were examined using standardized scores for the observed variables. Cases with absolute standardized scores greater than 3.29 were considered potentially extreme. Multivariate outliers were assessed using Mahalanobis distance with a significance criterion of *p* < 0.001. Nine cases were identified as potential multivariate outliers. Further inspection of their original responses and response patterns indicated that these cases represented plausible observations rather than data-entry errors or invalid responding. Sensitivity analyses were subsequently conducted by estimating the principal models both with and without the nine cases. The results produced substantively similar coefficient directions, significance patterns, and overall interpretations. The nine cases were therefore retained, and the final analytical sample remained 801 participants.

Descriptive statistics, including means, standard deviations, skewness, and kurtosis, were calculated for the observed variables. The distributional properties of the data were examined before model estimation. Pearson correlation analysis was also performed to describe the bivariate relationships among CSE, HAS, ALT, and perceived KAA.

Second, common method bias was evaluated using the procedures described in Section 3.3. Harman’s single-factor test was first conducted to determine whether a single factor accounted for most of the total variance. A single-factor confirmatory factor analysis model was then compared with the hypothesized four-factor measurement model. Finally, an unmeasured latent method construct was added to the measurement model to examine whether the inclusion of a general method factor substantially improved model fit or altered the interpretation of the substantive factor structure.

Third, confirmatory factor analysis was conducted using maximum likelihood estimation to evaluate the measurement model. Standardized factor loadings, Cronbach’s alpha, composite reliability (CR), and average variance extracted (AVE) were used to assess indicator reliability, internal consistency, and convergent validity. Cronbach’s alpha and CR values of at least 0.70 and AVE values of at least 0.50 were considered acceptable. Discriminant validity was assessed using the Fornell–Larcker criterion, according to which the square root of the AVE for each construct should exceed its correlations with the other constructs (Fornell and Larcker, 1981).

Fourth, structural equation modeling was used to test the hypothesized relationships among the four latent constructs. The structural model included paths from CSE to HAS, ALT, and perceived KAA, together with paths from HAS and ALT to perceived KAA. Because HAS and ALT represented related but conceptually distinct forms of AI-supported learning behavior, their residuals were allowed to covary. Model fit was evaluated using the chi-square statistic, degrees of freedom, the chi-square-to-degrees-of-freedom ratio, the goodness-of-fit index (GFI), comparative fit index (CFI), Tucker–Lewis index (TLI), root mean square error of approximation (RMSEA), and standardized root mean square residual (SRMR).

Finally, the indirect associations of CSE with perceived KAA through HAS and ALT were examined using bias-corrected bootstrapping with 5,000 resamples and 95% confidence intervals. Bootstrapping was employed because it does not require the sampling distribution of an indirect association to be normally distributed and provides robust confidence intervals for mediation analysis (Pesigan and Cheung, 2020). An indirect association was considered statistically significant when its 95% confidence interval did not include zero.

## 4 Results

### 4.1 Data normality and common method bias testing

Before evaluating the measurement and structural models, the distributional characteristics of the observed variables were examined. The absolute values of skewness ranged from 0.234 to 0.659, while the absolute values of kurtosis ranged from 0.332 to 1.300. Given the large sample size and the relatively small skewness and kurtosis values, the observed variables did not exhibit substantial univariate non-normality (Kim, 2013). Maximum likelihood estimation was therefore used in the subsequent confirmatory factor analysis and structural equation modeling.

Because all focal variables were collected from the same respondents through a self-reported questionnaire at a single time point, common method variance was assessed using three complementary statistical procedures. First, Harman’s single-factor test was conducted using all measurement items. Four factors with eigenvalues greater than 1 were extracted, jointly accounting for 84.072% of the total variance. The first unrotated factor explained 42.816% of the variance, which was below the commonly used 50% criterion. This result provided preliminary evidence that no single factor accounted for most of the covariance among the measurement items. However, because Harman’s single-factor test alone cannot conclusively exclude common method bias, two additional model-based examinations were performed (Podsakoff et al., 2003).

Second, a single-factor confirmatory factor analysis model was estimated and compared with the hypothesized four-factor measurement model. As shown in Table 2, the single-factor model demonstrated substantially poorer fit, *χ*^2^ = 4143.752, df = 54, than the hypothesized four-factor model, *χ*^2^ = 113.805, df = 48. The chi-square difference was statistically significant, Δ*χ*^2^ = 4029.947, Δdf = 6, *p* < 0.001. These results indicated that the four constructs were empirically distinguishable and that the covariance among the measurement items could not be adequately represented by a single general factor.

**Table 2:** Changes of CFA comparison model.

Model	*χ* ^2^	df	Δ*χ*^2^	Δdf	*p*
One-factor	4143.752	54	4029.947	6	<0.001
Four-factor	113.805	48			

The four-factor model was compared with the one-factor model. A significant chi-square difference supports the distinctiveness of the four constructs.

Third, an unmeasured latent method construct was introduced into the four-factor measurement model following the ULMC approach (Liang et al., 2007). The baseline four-factor model demonstrated satisfactory fit, *χ*^2^ = 113.805, df = 48, *χ*^2^/df = 2.371, GFI = 0.975, CFI = 0.991, TLI = 0.988, RMSEA = 0.041, and SRMR = 0.0217. After the general method factor was added, the model fit improved to *χ*^2^ = 43.478, df = 36, *χ*^2^/df = 1.208, GFI = 0.991, CFI = 0.999, TLI = 0.998, RMSEA = 0.016, and SRMR = 0.0126. The corresponding changes were ΔGFI = 0.016, ΔCFI = 0.008, ΔTLI = 0.010, ΔRMSEA = −0.025, and ΔSRMR = −0.009 Table 3.

**Table 3:** Results of the ULMC test.

Model	*χ* ^2^	df	*χ*^2^/df	GFI	CFI	TLI	RMSEA	SRMR	△GFI	△CFI	△TLI	△RMSEA	△SRMR
M1	113.805	48	2.371	0.975	0.991	0.988	0.041	0.0217	0.016	0.008	0.010	−0.025	−0.009
M2	43.478	36	1.208	0.991	0.999	0.998	0.016	0.0126					

Changes from M1 to M2 were ΔGFI = 0.016, ΔCFI = 0.008, ΔTLI = 0.010, ΔRMSEA = −0.025, and ΔSRMR = −0.009.

Although the addition of the method factor improved several model-fit indices, the changes in the incremental fit indices were modest, and the baseline four-factor model already showed satisfactory fit. The substantive four-factor structure therefore remained stable after the general method factor was introduced. Nevertheless, the improvement in overall fit indicates that some common method variance cannot be completely ruled out. Taken together, the three tests suggest that common method variance did not dominate the observed factor structure or invalidate the distinction among CSE, HAS, ALT, and perceived KAA, although a limited degree of method-related variance may have been present.

### 4.2 Descriptive statistics

Descriptive statistics for the four core constructs are presented in Table 4. The mean scores of all constructs were above the theoretical midpoint of 3 on the five-point Likert scale. Autonomous learning technology use had the highest mean score (*M* = 3.864, SD = 0.700), followed by perceived knowledge application ability (*M* = 3.802, SD = 0.814), human–AI synergy (*M* = 3.681, SD = 0.711), and computer self-efficacy (*M* = 3.673, SD = 0.808). Overall, participants reported above-midpoint levels of computer self-efficacy, human–AI synergy, autonomous learning technology use, and perceived knowledge application ability.

**Table 4:** Descriptive statistics.

Dimension	Mean	SD
ALT	3.864	0.700
CSE	3.673	0.808
HAS	3.681	0.711
KAA	3.802	0.814

*N* = 801. All constructs were measured using a five-point Likert scale ranging from 1 (strongly disagree) to 5 (strongly agree). SD, standard deviation; ALT, autonomous learning technology use; CSE, computer self-efficacy; HAS, human–AI synergy; KAA, perceived knowledge application ability.

### 4.3 Measurement model: reliability and validity

Confirmatory factor analysis was conducted to evaluate the reliability and validity of the measurement model. As shown in Table 5, all standardized factor loadings ranged from 0.629 to 0.932, indicating acceptable to excellent item-level representation of the latent constructs. Cronbach’s alpha values ranged from 0.814 to 0.949, and composite reliability values ranged from 0.821 to 0.949. These values exceeded the recommended threshold of 0.70, demonstrating strong internal consistency.

**Table 5:** Reliability and convergent validity of the measurement model.

Constructs	Indicators	*B*	SE	Z	*p*	*β*	Cronbach’s α	CR	AVE
KAA	KAA1	1				0.887	0.949	0.949	0.823
	KAA2	1.028	0.026	40.153	***	0.919			
	KAA3	1.033	0.025	41.484	***	0.932			
	KAA4	0.996	0.027	37.163	***	0.889			
HAS	HAS1	1				0.629	0.814	0.821	0.608
	HAS2	1.204	0.066	18.211	***	0.825			
	HAS3	1.249	0.067	18.618	***	0.865			
CSE	CSE1	1				0.919	0.909	0.908	0.831
	CSE2	0.974	0.030	32.523	***	0.904			
ALT	ALT1	1				0.881	0.903	0.903	0.755
	ALT2	1.004	0.030	33.092	***	0.882			
	ALT3	0.948	0.031	30.881	***	0.844			

*** *p* < 0.001; CR, composite reliability; AVE, average variance extracted.

Convergent validity was assessed using AVE. The AVE values ranged from 0.608 to 0.831, all exceeding the recommended threshold of 0.50. This suggests that each construct explained a sufficient proportion of variance in its indicators. Discriminant validity was evaluated using the Fornell-Larcker criterion. As shown in Table 6, the square root of the AVE for each construct was greater than its correlations with other constructs, indicating adequate discriminant validity.

**Table 6:** Discriminant validity.

Variable	ALT	CSE	HAS	KAA
ALT	**0.869**			
CSE	0.676	**0.912**		
HAS	0.708	0.733	**0.780**	
KAA	0.237	0.140	0.054	**0.907**

Diagonal values are the square roots of AVE. Off-diagonal values are Pearson correlations.

### 4.4 Structural model and hypothesis testing

The final structural model showed acceptable global fit: *χ*^2^ = 103.867, df = 47, *χ*^2^/df = 2.210, GFI = 0 0.978, AGFI = 0 0.963, CFI = 0.992, TLI = 0.989, RMSEA = 0.039, and SRMR = 0.0191.

As shown in Table 7, CSE was positively associated with HAS (*B* = 0.547, SE = 0.024, z = 22.467, *p* < 0.001, *β* = 0.622), supporting H2. CSE was also positively associated with ALT (*B* = 0.528, SE = 0.024, *z* = 21.774, *p* < 0.001, *β* = 0.610), supporting H3. The direct association between CSE and perceived KAA was not significant after the mediators were included (*B* = 0.052, SE = 0.052, *z* = 1.011, *p* = 0.312, *β* = 0.051); therefore, H1 was not supported at the direct-path level.

**Table 7:** Path coefficients and hypothesis testing results of the structural model.

Path	B	SE	*z*	*p*	*β*	Decision
H2: CSE → HAS	0.547	0.024	22.467	***	0.622	Supported
H3: CSE → ALT	0.528	0.024	21.774	***	0.610	Supported
H1: CSE → KAA	0.052	0.052	1.011	0.312	0.051	Not supported
HAS→KAA	−0.158	0.050	−3.152	0.002	−0.137	Significant
ALT→KAA	0.314	0.050	6.243	***	0.268	Significant

*** *p* < 0.001; *B* = unstandardized coefficient; *β* = standardized coefficient.

HAS showed a small negative conditional association with perceived KAA (*B* = −0.158, SE = 0.050, z = −3.152, *p* = 0.002, *β* = −0.137), whereas ALT showed a positive conditional association with perceived KAA (*B* = 0.314, SE = 0.050, *z* = 6.243, *p* < 0.001, *β* = 0.268). The disturbances of HAS and ALT were allowed to covary; the corresponding standardized residual association was approximately 0.424 after accounting for CSE, indicating shared variance not explained by CSE.

### 4.5 Mediation effect testing

The indirect associations were examined using bias-corrected bootstrapping with 5,000 resamples. As shown in Table 8, the indirect association between CSE and perceived KAA through HAS was negative and significant, supporting H4. The indirect association through ALT was positive and significant, supporting H5.

**Table 8:** Parallel indirect associations through HAS and ALT.

Path relationship	Estimate	95% CI (lower)	95% CI (upper)	Decision
CSE → HAS→KAA(IE1)	−0.087	−0.156	−0.023	Supported
CSE → ALT→KAA(IE2)	0.166	0.097	0.235	Supported
CSE → KAA(DE)	0.052	−0.038	0.142	Not significant
Total = (IE1 + IE2 + DE)	0.132	0.052	0.209	Significant

Confidence intervals that do not include zero indicate significant effects.

The direct association between CSE and perceived KAA was not significant after HAS and ALT were included, whereas the total association remained positive and significant. The two indirect associations therefore operated in opposite directions, with the positive indirect association through ALT being larger than the negative indirect association through HAS. Accordingly, H6 was supported at the level of parallel mediation. These findings represent conditional indirect associations and do not establish causal mechanisms or temporal precedence.

### 4.6 Robustness and interpretive checks

The standardized paths implied that CSE accounted for approximately 38.7% of the variance in HAS and 37.2% of the variance in ALT. The three predictors together accounted for approximately 4.9% of the variance in perceived KAA. The comparatively low explained variance for perceived KAA indicates that most variation in the outcome was associated with factors outside the present model and requires restrained interpretation of practical importance.

Construct-level collinearity diagnostics based on the predictor correlation matrix yielded VIF values of approximately 2.42 for CSE, 2.63 for HAS, and 2.24 for ALT. These values did not indicate severe multicollinearity, but the relatively strong correlations among CSE, HAS, and ALT mean that each structural coefficient is conditional on the other predictors. This point is especially important for HAS. Its zero-order correlation with perceived KAA was 0.054, whereas its standardized path coefficient became −0.137 after CSE and ALT were included. The negative path should therefore be interpreted as a conditional or suppressor-type association rather than evidence that HAS is inherently detrimental. The HAS measure did not assess cognitive offloading, and the design cannot determine whether students with lower perceived KAA seek more collaborative AI support or whether unmeasured variables influence both constructs. Alternative causal orderings cannot be adjudicated by the present covariance structure. A reverse ordering can be covariance-equivalent to the hypothesized ordering in cross-sectional SEM, so global fit does not establish temporal precedence. The hypothesized direction is retained because it is consistent with Social Cognitive Theory, but it remains an explanatory specification rather than a causal test.

## 5 Discussion

### 5.1 Summary of findings

This study examined the association between CSE and perceived KAA through HAS and ALT among 801 undergraduate students from five higher education institutions in Shandong Province. CSE was positively associated with both forms of AI-related learning behavior. ALT showed a positive conditional association with perceived KAA, whereas HAS showed a small negative conditional association when CSE and ALT were simultaneously included in the model. The two indirect associations were statistically significant and operated in opposite directions, while the direct association between CSE and perceived KAA was not significant after the mediators were included. Overall, the findings indicate that CSE is associated with perceived knowledge application through distinct patterns of AI-supported learning behavior.

### 5.2 Computer self-efficacy and AI-related learning behaviors

The positive associations of CSE with HAS and ALT are consistent with Social Cognitive Theory. Students who believe they can use digital technologies effectively may be more willing to initiate AI interaction, sustain multi-turn dialogue, and manage technical difficulties. They may also be more likely to integrate AI tools into self-directed routines for planning, resource searching, and monitoring learning. These findings extend research linking self-efficacy to technology adoption by showing that confidence is related to more than a general intention to use AI; it is associated with multiple modes of learning behavior (Alghamdi, 2025; Lin and Wang, 2025). The similar magnitudes of the CSE-HAS and CSE-ALT paths suggest that technological confidence may support both interactive collaboration and autonomous regulation. These behaviors should not be treated as mutually exclusive. A confident student may engage deeply with an AI system while also maintaining control over goals, standards, and final decisions. Future models should examine how the quality of regulation shapes the relationship between collaboration and autonomy.

### 5.3 Autonomous learning technology use and perceived knowledge application

ALT was positively associated with perceived KAA and carried the larger positive indirect association. This result is consistent with research emphasizing learner agency and self-directed technology integration. Students who use AI tools to plan learning, organize resources, monitor understanding, and support independent problem solving may experience greater control over the learning process and may consequently report stronger confidence in applying knowledge (Lin et al., 2025; Ge and Li, 2026).

The finding also aligns with emerging research on AI-supported self-regulated learning and learner autonomy. Strategic use requires learners to determine when assistance is appropriate, evaluate output quality, connect information to prior knowledge, and revise goals or strategies. Metacognitive regulation may therefore be an important unmeasured mechanism linking ALT to perceived KAA (He, 2025; El Hosayny et al., 2026; Wu et al., 2026). Because these regulatory processes were not directly assessed, the present study cannot determine which components of ALT were most influential.

### 5.4 Interpreting the negative conditional association of human-AI synergy

The HAS result requires particular caution. HAS had an almost zero bivariate correlation with perceived KAA (*r* = 0.054), but its coefficient became negative when CSE and ALT were included. This pattern is consistent with a suppression or conditional association among correlated predictors. It does not support the simple statement that more human-AI synergy is associated with lower perceived KAA at the zero-order level. Several explanations remain possible. First, when the self-regulatory component shared with ALT is held constant, the remaining variance in HAS may represent reliance on interactive support that is not accompanied by autonomous regulation. Second, students who feel less capable of applying knowledge may seek more intensive AI collaboration, producing reverse selection. Third, unmeasured factors such as metacognitive regulation, AI literacy, critical thinking, prior achievement, discipline, and actual AI experience may influence both collaboration patterns and perceived KAA. Fourth, the negative coefficient may partly reflect statistical suppression created by the strong correlations among CSE, HAS, and ALT. Cognitive offloading is one theoretically plausible issue for future study, particularly where learners delegate reasoning, evaluation, or problem solving to AI (Fan et al., 2025; Zahid et al., 2026). Nevertheless, it was not directly measured here. The HAS items describe task division and collaboration, which may be productive when learners question outputs, compare alternatives, and maintain responsibility for decisions (Hou et al., 2025; Wong and Qiu, 2026). Future research should distinguish productive synergy from dependent synergy using separate measures of AI reliance, cognitive offloading, verification behavior, and metacognitive monitoring.

### 5.5 Theoretical implications

First, this study extends Social Cognitive Theory to GAI-supported learning by showing that computer self-efficacy is associated with perceived knowledge application through multiple behavioral pathways. The findings support the theoretical importance of behavioral enactment while also indicating that cross-sectional mediation coefficients should be interpreted as associations rather than causal processes.

Second, the study distinguishes HAS from ALT. Much of the AI-in-education literature treats AI use as a broad or homogeneous construct, potentially obscuring differences between interactive collaboration and self-directed regulation. Although HAS and ALT were positively correlated, their conditional associations with perceived KAA differed when they were modeled simultaneously. This finding supports a more differentiated conceptualization of AI-supported learning behavior.

Third, the results demonstrate the importance of considering zero-order correlations, shared variance, and correlated predictors when interpreting parallel mediation models. The change in the HAS coefficient after CSE and ALT were simultaneously included indicates that conditional structural coefficients should not be interpreted as simple bivariate relationships or as direct evidence of an underlying psychological mechanism.

Finally, the relatively small proportion of explained variance in perceived KAA suggests that technological confidence and AI-use behavior constitute only part of a broader system. Prior knowledge, disciplinary demands, task design, feedback quality, metacognitive ability, motivation, and assessment conditions are likely to explain additional variation. Future theoretical models should integrate these factors rather than treating AI-related behavior as a sufficient explanation of perceived learning outcomes.

### 5.6 Practical implications

The results do not justify discouraging human-AI collaboration. Instead, institutions should focus on the quality and regulation of collaboration. AI literacy programs can teach students to formulate questions, verify claims, compare sources, document revisions, and explain why AI-generated suggestions were accepted or rejected. These practices support accountability without assuming that interaction itself is harmful.

Instructors can design activities that combine AI assistance with visible learner reasoning. Students may be asked to produce an initial analysis before consulting AI, annotate changes made after feedback, identify errors or unsupported claims in generated content, and provide reflective explanations of final decisions. Such designs can help preserve cognitive participation and make metacognitive regulation observable.

Assessment should distinguish supported task completion from independent knowledge application. Oral defenses, transfer problems, process portfolios, reflective commentaries, and selected AI-free components can complement conventional written products. These approaches are especially relevant because the outcome in the present study was perceived KAA rather than demonstrated performance.

Finally, educators should avoid treating either more interaction or more autonomy as universally superior. The appropriate balance may depend on task complexity, discipline, students’ prior knowledge, and the reliability of available AI tools. Guidance should help students decide when collaboration is beneficial, when independent effort is needed, and how responsibility for learning outcomes remains with the learner.

### 5.7 Limitations and future research

Several limitations should be considered. First, the cross-sectional design does not establish temporal precedence or causal relationships. Although the proposed ordering was informed by Social Cognitive Theory, reverse or reciprocal associations remain possible. Future research should employ longitudinal or experimental designs to examine changes in computer self-efficacy, AI-supported learning behavior, and knowledge application over time. Second, all focal constructs were assessed using self-reported measures. Perceived KAA should not be interpreted as objectively demonstrated knowledge transfer, and the HAS measure assessed perceived collaboration rather than cognitive offloading, dependence, or verification behavior. In addition, the final CSE measure contained only two items after one item was removed during measurement evaluation. Future studies should combine self-reports with performance-based transfer tasks, learning analytics, AI interaction records, and more comprehensive measures of AI-related self-efficacy and collaboration. Third, the questionnaire followed a fixed sequence rather than a randomized or counterbalanced order. Potential order, priming, and fatigue effects therefore cannot be ruled out. Future surveys should use randomized scale blocks or balanced questionnaire versions where appropriate. Fourth, participants were recruited through convenience and snowball sampling from five higher education institutions in one Chinese province. The sample should therefore not be considered representative of all Chinese undergraduate students. Multi-region, stratified, or probability-based sampling would improve the generalizability of future findings. Finally, demographic characteristics and patterns of generative AI use were collected for descriptive purposes but were not included as covariates or moderators in the structural model. Moreover, the predictors explained only a limited proportion of variance in perceived KAA. Future studies should examine the roles of academic discipline, AI-use experience, prior achievement, AI literacy, critical thinking, motivation, metacognitive regulation, and task characteristics.

## 6 Conclusion

This cross-sectional study examined how CSE was associated with perceived knowledge application through HAS and ALT. CSE was positively associated with both AI-related learning behaviors. ALT showed a positive indirect association with perceived KAA, whereas HAS showed a negative conditional indirect association when the correlated predictors were simultaneously modeled. The direct association between CSE and perceived KAA was not significant after the mediators were included, while the total association remained positive. Overall, the findings support a differentiated view of GAI-supported learning and suggest that the educational relevance of AI use may depend on how students combine interactive collaboration with autonomous regulation. Because perceived KAA was measured through self-report and the data were cross-sectional, the findings should be interpreted as associations rather than causal effects. Research and practice should therefore focus on how students regulate AI collaboration, maintain responsibility for reasoning, and integrate technological assistance into autonomous learning processes.

## Data Availability

The raw data supporting the conclusions of this article will be made available by the authors, without undue reservation.
