# Supplementary material for: Computer self-efficacy and perceived knowledge application in generative AI-supported learning: a cross-sectional parallel mediation analysis
Source: Front Psychol. 2026 Aug 26;17:1927402. doi: 10.3389/fpsyg.2026.1927402 (PMC13553910; doi:10.3389/fpsyg.2026.1927402)
Supplement: Supplementary file 1 [file Data_Sheet_1.PDF]

Supplementary Table S1. Measurement Items, Sources, and Retention Status

| Construct | Item code | Full item | Adapted from | Status |
|---|---|---|---|---|
| CSE | CSE1 | I am confident in independently solving technical problems encountered when using AI tools for learning. | Fang et al. (2024) | Retained |
| CSE | CSE2 | I am confident that I can learn to use unfamiliar functions of AI tools for academic learning tasks. | Fang et al. (2024) | Retained |
| CSE | CSE3 | Even when an AI tool does not work as expected, I am confident that I can adjust my approach and continue the learning task. | Fang et al. (2024) | Removed |
| HAS | HAS1 | When completing complex learning tasks, I can form effective task division and collaboration with AI tools. | Li et al. (2023) | Retained |
| HAS | HAS2 | I engage in repeated dialogue with AI tools to develop and refine ideas during learning. | Li et al. (2023) | Retained |
| HAS | HAS3 | I combine my own reasoning with suggestions provided by AI so that they play complementary roles in completing learning tasks. | Li et al. (2023) | Retained |
| ALT | ALT1 | I actively use AI technology to plan and manage my extracurricular learning progress. | Teo et al. (2010) | Retained |
| ALT | ALT2 | I use AI tools to monitor my understanding and adjust my learning strategies when necessary. | Teo et al. (2010) | Retained |
| ALT | ALT3 | When I encounter a learning problem, I independently decide how to use AI to locate relevant information and support problem solving. | Teo et al. (2010) | Retained |
| KAA | KAA1 | I can flexibly apply new knowledge acquired through AI assistance to actual course projects or problem solving. | Lo et al. (2022) | Retained |
| KAA | KAA2 | I can adapt newly acquired knowledge to new or unfamiliar academic situations. | Lo et al. (2022) | Retained |
| KAA | KAA3 | I can integrate knowledge and information from different sources to develop a coherent solution to a problem. | Lo et al. (2022) | Retained |
| KAA | KAA4 | When facing a complex task, I can draw on what I have learned to guide my decisions and actions. | Lo et al. (2022) | Retained |

***Note.*** *All items were rated on a five-point Likert scale ranging from 1 (strongly disagree) to 5 (strongly agree). CSE = computer self-efficacy; HAS = human–AI synergy; ALT = autonomous learning technology use; KAA = perceived knowledge application ability. The items were adapted and contextualized for generative AI-supported learning. One CSE item was removed during measurement-model evaluation because it did not demonstrate*

*adequate psychometric performance. The final measurement model contained 12 retained items.*
